# Research on the Use of Silicon-Ethanol Composite in Actuators

**DOI:** 10.3390/ma15238570

**Published:** 2022-12-01

**Authors:** Tomasz Kapłon, Andrzej Milecki

**Affiliations:** Institute of Mechanical Technology, Poznan University of Technology, Pl. M. Skłodowskiej-Curie 5, 60-965 Poznan, Poland

**Keywords:** silicon-ethanol composite, phase-change material actuators, smart materials, actuators design

## Abstract

Silicon-ethanol is a relatively new smart composite in the category of phase-change materials (PCM). It consists of liquid ethanol entrapped in bubbles spread into a silicone rubber matrix, i.e., during cooling. The composite is able to expand significantly when heat is applied and shrink when it is removed. The properties of this material can be used in a new type of actuator. In this paper, the basic equations that describe the properties of actuators with a silicon-ethanol composite are given. Using them, two solutions of unidirectional actuators with a composite inserted into polycarbonate tubes and metal bellows are designed and investigated. In the study, actuators with different geometric dimensions and applied composite volumes are investigated. The elongations of the actuators and the blocking forces are measured. The theoretical relationships given at the beginning of the paper that describe the properties of the composite are validated using the performed experimental results of the built actuators. The tube actuators achieved elongation between 32% and 35% at a temperature of 75 degrees Celsius, that is, less than that predicted according to equations from earlier publications. Due to this, a modified equation that includes the influence of friction was proposed and compared with experimental results. The performance of the tube actuator deteriorates rapidly. In the case of bellow actuators, they stabilize after a few cycles of heating and cooling.

## 1. Introduction

The concept of applying phase state change materials in actuators was proposed many years ago. The most well-known example is paraffin actuators, commonly used as thermally activated actuators [1]. They use the phenomenon of change in the specific volume of the active medium as a result of a change in its physical state. In the case of paraffin in solid form, a reversible solid–liquid transformation occurs as a result of an increase or decrease in the temperature. There are also other examples of the use of liquids as an active medium, for example, water or alcohol controlled by temperature actuators [2,3,4]. In these cases, a reversible gas–liquid transition takes place.

In recent years, the concept of using composite materials in which the active medium is surrounded by an elastic matrix has been applied in special actuators. One such material is a silicone-paraffin composite, consisting of paraffin mixed with a silicone rubber matrix [5]. The concept of composites consisting of liquids placed in an elastic matrix has also been proposed. As a matrix material, silicone rubber is usually used. As the active medium, a liquid with a relatively low boiling point, most often ethanol, is applied. There were also concepts of actuators in which the matrix material was a mixture of silicon and liquid metal [6]. In other solutions, the actuator consisted of a relatively large tank made of flexible material, such as silicone rubber mixed with liquid alcohol. However, in such cases, the main problem was the durability of the actuator performance due to the rapid escape of the active medium, that is, alcohol, which is usually very volatile [7].

The silicone–ethanol composite is the next example of materials used in actuators. One of the first uses of this material is described in the paper Soft material for soft actuators [8]. The composite consists of a matrix of silicone rubber and ethanol enclosed in bubbles within the matrix. Casting is the most common method to produce a silicone–ethanol composite. The possibility of using 3D-FDM (additive manufacturing) printing of this composite has also been demonstrated [9]. In the case of casting silicone–ethanol composites, unlike casting conventional silicones, deaeration is not used to prevent ethanol from escaping from the material. After production, the silicone matrix contains bubbles filled with liquid ethanol and a mixture of ethanol vapors and air. The typical complex cycle of such composite work consists of gradual heating, during which the actuator extends, and then free cooling, in which it shrinks. In the first phase, a liquid–gas transition begins after the boiling point of ethanol is reached, that is, at approximately 78.4 °C at atmospheric pressure [8]. Parts of the ethanol, changing the state of aggregation from liquid to gas, significantly increase their inner pressure, causing an increase in specific volume, i.e., in the expansion of the bubbles, and thus the elongation of the actuator is obtained. The stretching of the bubbles is counteracted by the elastic forces generated by the elastic matrix, which simultaneously results in an increase in the boiling point and the cessation of the liquid–gas transformation process. For this reason, the condition for the continuation of the transformation is the further heating of the composite, whereby the ethanol remaining in the bubbles may change its phase to gas, causing the composite to expand further. If all the liquid ethanol is converted into gas, further expansion of it can take place only by increasing the volume of the heated ethanol vapor. During cooling, the reverse process takes place, i.e., cooled ethanol returns to the liquid state. In this process, the elastic forces of the matrix, which are not counteracted by the pressure inside the bubbles, cause the material to return to its original dimensions [8]. These changes are reflected in the deformation of the actuator, i.e., its shortening as a result of temperature changes. A significant problem with the composite described above is the loss of ethanol over time. This is due to the diffusion of ethanol vapors through the silicone into the surrounding atmosphere. This process significantly accelerates with an increasing temperature; therefore, the temperature in the vicinity of saturation not only results in a slight increase in deformation but also accelerates actuator wear and deterioration of its performance due to leakage of ethanol [10]. To reduce the escape of ethanol, it was suggested in the literature [11] that additional sealing or covering of the composite should be applied. Typically, an additional outer impermeable layer is added that insulates against the outer atmosphere. However, no research has been performed on the effectiveness of this method so far [9]. Because ethanol can also diffuse within the composite, it is possible to regenerate the actuators. This can be achieved by immersing the composite in ethanol and leaving it in such a bath for the time needed for regeneration. In some proposed manufacturing processes for composite-based actuators, the preformed composite is even used to evaporate some of the ethanol and then soak it again [10].

In all actuators proposed so far with silicone-ethanol, the temperature of the composite was increased by the conduction of heat to it through an electric heating element. The most common solution is to use the heater in the form of a spiral made of resistance wire embedded within the compound [9,11]. A typical heater is made of thin wire and has the form of a spring, thanks to which it can extend along with the expanding composite. A certain disadvantage of this solution is that the composite directly adjacent to the resistance wire heats up strongly, thus losing the ethanol, and degenerating significantly. This is because of the low thermal conductivity of the base material. Another concept was to make the heater in the form of a core of a flexible conductive material that forms a monolithic whole with the composite [12]. It was made of silicone mixed with a conductive material such as graphite or carbon. Such a material has the ability to conduct electricity and is characterized by high resistance because it can emit a significant amount of heat. Because it is silicone-based, it could move, i.e., expand with the rest of the composite. Furthermore, this type of heater could be formed in a shape that increases the conductive surface [12]. The use of a special conductive fabric was also proposed for use in the actuator described here [13]. As a result, an increase in the conductive area was achieved between the heating element and the composite. In previous work [14], a different method was proposed for the heating of the composite in an actuator, for which the heating was achieved indirectly by induction. In this case, the composite was enclosed in a metal casing in the form of an aluminum cylinder that was heated with induction. In this way, an increase in the conductive area between the heating element and the composite was achieved.

Various forms of actuators using a silicone-ethanol composite are described in [9]. In simple unidirectional actuators, a composite is enclosed in a cylindrical housing with a movable piston. If the composite is heated, it extends axially and thus pushes a piston in a linear motion. In most of the cases described in the literature of such actuators, one-way movement during expansion was primarily investigated. A significant disadvantage of this solution is the presence of friction between the cylinder wall and the composite, which disrupts its movement. Another serious problem is obtaining the return movement, which can be induced as a result of composite cooling. The review of the literature showed that in previous research, only natural cooling was used, consisting of the free transfer of heat from the composite housing to the surrounding atmosphere at room temperature. As a result, the cooling process was not controlled and took much longer than the heating process; thus, the return time of the actuators was also significantly longer than the extension time. The return movement is particularly susceptible to changes in ambient temperature and is therefore difficult to control. Therefore, the cylinder actuators described above are actually only considered one-way solutions.

Unidirectional linear and bending artificial muscles based on composite silicon-ethanol [8,11]. They are inspired by the concept of pneumatic artificial muscles and are modeled on McKibben actuators. The difference between composite-based muscles and pneumatic muscles is that in the braid, instead of balloons filled with compressed air, a composite is placed with a heater inside. The expansion of the heated composite increases the diameter of the braid, resulting in a shortening of its length that mimics the contractile response of the muscles. Such muscles can perform a pulling motion directly. Return movement, i.e., pushing, is caused by the elastic balloon and the braid [9,11] when the composite temperature decreases.

The actuator in the form of a metal bellows filled with composite works similarly to a unidirectional push motion muscle [15]. In this case, the problem of friction between the composite and the cylinder walls does not exist. The metal bellows give the actuator the stiffness required to perform the pushing motion without buckling. In addition, the spring force of the bellows allows for return movement. The disadvantage of the composite muscles is that the elasticity of the bellows limits the maximum achievable displacement. In turn, the advantage of using bellows is that, because of their ribbing, they facilitate heat dissipation.

In the literature, composite-based actuators in which the bending movement is performed are also proposed and described in [16,17]. So far, designers of these solutions are looking for applications in the field of flexible robots, called soft robots.

The greatest weakness of actuators based on silicone–ethanol composites is their low speed of operation [18]. This is because the bases of their functioning are thermal processes of heating and cooling that are usually long-term. The possibilities of improving the dynamics consist of accelerating the heating and cooling process. The attempt to improve both aspects by introducing additives to the composite that increase its conductivity is proposed in [18,19]. An improvement in the heating speed can also be achieved by modifying the heating methods.

## 2. Theoretical Background

The theoretical proposition of a model that describes the behavior of the heated composite was proposed in the article [16]. The factor that influences the expansion of the composite is the internal pressure *p* that increases in the ethanol vapor pressure caused by the increase in temperature. The vapor pressure is the pressure for saturated vapor at which the gas is in equilibrium with the liquid at a given temperature. The ethanol vapor pressure for a temperature *T* can be calculated using the experimental equation given in [16,20]. If the temperature of ethanol changes over time, the equation that describes the vapor pressure *p*(*t*) can be described as follows:

(1) $$p\left(t\right)=p_{c}e^{\frac{T_{C}}{T\left(t\right)}\left(-0.051477\theta^{1/2}-8.27075\theta -5.49245\theta^{3}+5.64829\theta^{11/2}\right)}$$

(2) $$\theta \left(t\right)=1-\frac{T\left(t\right)}{T_{C}}$$

where *T*—temperature of ethanol in Kelvin, *T_C_*—critical temperature of ethanol equal to 513.9 K, *p*_c_—critical pressure of ethanol equal to 6150 kPa, and *p*(*t*)*—*the pressure of ethanol (changes over time).

**Figure 1:**
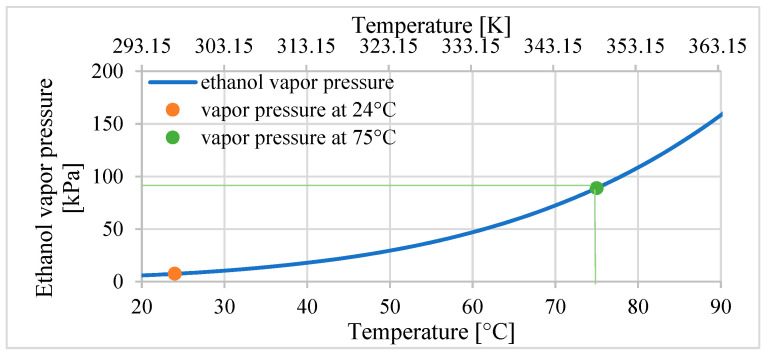
Vapor pressure of ethanol calculated using Equations (1) and (2).

This equation links the vapor pressure to parameters of the critical temperature *T_C_* of the ethanol and the critical pressure *p_C_*, which describe the critical point for the ethanol. It is the end point at the temperature–pressure curve that describes the conditions of coexistence of the liquid and gas states. According to Equations (1) and (2), the dependencies between the temperature and the vapor pressure of ethanol in the temperature range of 20 °C–90 °C are presented in Figure 1.

The internal pressure of the ethanol increases in the silicon–ethanol composite in the actuator based on it during temperature changes:

(3) $$\Delta p\left(t\right)=p\left(T\left(t\right)\right)-p_{0}\left(T_{0}\right)$$

where *p*_0_ (24 °C), equal to 7.4 kPa, is the vapor pressure of ethanol for the initial temperature *T*_0_, which is room temperature at approximately 24 °C.

The properties of the silicon–ethanol composite result from the combination of properties of the silicon matrix and the influence of bubbles filled with ethanol. In the case of thermal expansion, these bubbles can be treated as sources of additional internal pressure, while in the case of calculated bulk and sheer modulus, these bubbles can be treated as voids. For this reason, the material parameters of the silicon–ethanol composite are described as effective.

The authors of a previous paper [16] theoretically derived the following formulas describing the behaviors of the composite:

- The variable effective coefficient of thermal expansion:

(4) $$\alpha_{e}\left(t\right)=\frac{\kappa -\kappa_{e}}{3\kappa \kappa_{e}}\cdot \frac{\Delta p\left(t\right)}{\Delta T\left(t\right)}+\alpha =\frac{3\kappa +4\mu }{12\kappa \mu }\cdot \frac{f}{1-f}\cdot \frac{\Delta p\left(t\right)}{T\left(t\right)-T_{0}}+\alpha ,$$

- The effective constant bulk module of the silicon-ethanol composite

(5) $$\kappa_{e}=\left(1-f\right)\frac{4\mu }{3f\kappa +4\mu }\kappa ,$$

- The effective constant sheer module of the silicon-ethanol composite

(6) $$\mu_{e}=\left(1-f\right)\frac{9\kappa +8\mu }{9\kappa +8\mu +6f\left(\kappa +2\mu \right)}\mu ,$$

where:

*κ*—the bulk module of the silicon matrix for Ecoflex-00-03 equals 423 kPa.

*μ—*the sheer module of the silicon matrix for Ecoflex-00-03 equals 21.6 kPa.

*α*—coefficient of thermal expansion of the silicon matrix for Ecoflex-00-03

equal to 0.000284 K^−1^.

*f*—ethanol volume fraction of ethanol in silicon–ethanol composite (typically 0.2).

*κ_e_*—effective bulk module of silicon–ethanol composite (for composite with ethanol volume fraction *f* = 0.2*κ_e_* = 85.9 kPa).

*μ_e_*—effective sheer module of silicon–ethanol composite (for composite with ethanol volume fraction *f* = 0.2*μ_e_* = 15.2 kPa).

*α_e_*—effective coefficient of thermal expansion of silicon–ethanol composite K^−1^.

*ΔT*(*t*)—increase in temperature from 24 °C, *ΔT*$\left(t\right)=T\left(t\right)-T_{0}$.

The same authors proposed a constitutive equation describing the stress–strain relationship for the composite (7):

(7) $$\sigma =\left(\kappa_{e}-\frac{2}{3}\mu_{3}\right)tr\left(\varepsilon \right)I+2\mu \varepsilon -3\kappa_{e}\alpha_{e}\Delta TI$$

where:

σ and ***ε*** are stress and strain tensors.

***I***—identity tensor.

Solving Equation (7) for the circumferential interface for the tube with free movement along the axial direction defined as *z* and the radial direction defined as *r* with boundary conditions *σ_zz_* = 0 and *ε_rr_* = 0, the equation for free thermal expansion along the axial direction is obtained:

(8) $$\varepsilon_{zz}\left(t\right)=\frac{3\kappa_{e}\alpha_{e}\left(t\right)\Delta T\left(t\right)}{\kappa_{e}+4\mu_{e}/3}=\left[1-\frac{3f\left(3\kappa -4\mu \right)}{3\left(3+5f\right)\kappa +8\mu }\right]\frac{9\kappa }{3\kappa +4\mu }\alpha_{e}\left(t\right)\Delta T\left(t\right),$$

After the transformation of Equation (8), the absolute variable value of elongation can be calculated using the following equation:

(9) $$\Delta l\left(t\right)=l_{0}\cdot \varepsilon_{zz}\left(t\right)=\frac{3\kappa_{e}\alpha_{e}\left(t\right)l_{0}}{\kappa_{e}+4\mu_{e}/3}\cdot \Delta T\left(t\right),$$

where:

*ε_zz_*—free thermal expansion along the axial direction.

*l*_0_—initial length.

*Δl*—increase in length.

The same authors also proposed equitation for blocking stress at the two ends of the confined cylindrical composite as follows. It was obtained for the boundary conditions *ε_zz_ =* 0 and *ε_rr_* = 0

(10) $$\sigma_{zz}\left(t\right)=\left(\kappa_{e}+\frac{4}{3}\mu_{e}\right)\varepsilon_{b}-3\kappa_{e}\alpha_{e}\left(t\right)\Delta T\left(t\right)$$

where:

*σ_zz_*—blocking stress (*F_b_*/*A*), where *F_b_* is the blocking force.

In this equation, *ε_b_* = *Δl_b_*/*l*_0_ refers to relative expansion in which the composite is blocked and *Δl_b_* is an elongation in which the external force blocks expansion. The first component describes the influence of the elongation of the composite on stress. The second component describes the linear stress changes from changes in temperature. This equation is applicable only if *Δl_b_* is smaller than the maximum possible elongation *l_max_.* If the composite has no possibility to elongate, which means that *Δl_b_ =* 0 and *ε_b_ =* 0, the whole second component of Equation (10) is also equal to 0. In this case, the stress is described only by the first component.

The given above equation for free thermal expansion is proposed, only according to theoretical considerations, without any experimental confirmation, which is an important disadvantage.

The above considerations were carried out on the macroscale. For the application of the composite in systems on the micro- or nanoscale, these considerations would require elaboration. For macroscale systems, structures, and materials, their mechanical properties can be aptly described by classical continuum mechanics. However, in the case of the nanoscale, this approach cannot describe the unique effects occurring at this small scale [21,22]. This is because in the constitutive relations of classical continuum mechanics, the internal characteristic scales of the material are not included. However, the influences of internal and external characteristic scales at the nanoscale are at a similar level, and both must be included in the analyses. Several methods for investigating mechanical properties on the nanoscale have been proposed, such as generalized continuum theories, molecular dynamics, atomic simulations, and nanoscale experiments. The most commonly used generalized continuum theories are, however, nonlocal theory and strain gradient theory [21,22].

## 3. Materials and Methods

For the design, construction, and investigation of actuators based on silicon-ethanol material, the authors of this paper prepared a silicon–ethanol composite. As a composite matrix, the type Ecoflex 00-03 silicon material was used. This is a two-component silicone, and it solidifies after mixing its two components in a 1:1 ratio. This silicon is commonly used in soft robotics applications. The active material is prepared as a composite consisting of ethanol as the active material and silicon, which was used as the matrix. We decided to select the share of ethanol and silicone in the material tested in preliminary studies. The ethanol was commercially available at 99.9% purity. The silicon parameters used are as follows: Young’s module of 0.07 MPa, a density of 1.07 g/cm^3^, a tensile strength of 1.38 MPa [23], a bulk module of 423 kPa, a sheer module of 21.6 kPa, and a coefficient of thermal expansion (*CTE*) of 284.2 × 10^−6^ K^−1^ [24]. *CTE* binds the thermal strain $\left(\varepsilon_{T}\right)$ with an increase in the temperature *ΔT* with the simple relation $\varepsilon_{T}=CTE\cdot \Delta T$.

**Figure 2:**
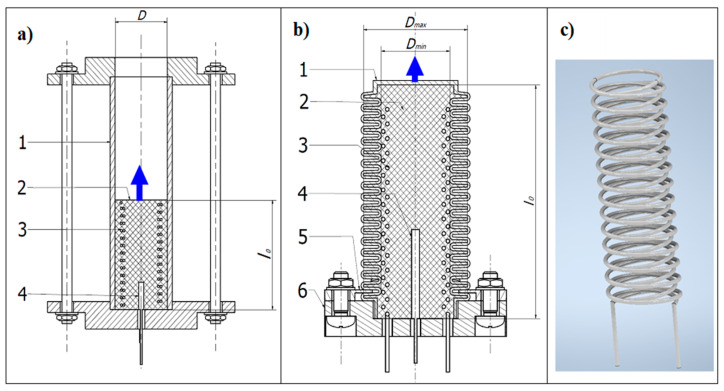
Construction of investigated unidirectional actuators: (**a**) Tube actuator where 1—polycarbonate tube, 2—composite 3—heater, i.e., spring-like wires—double coil, 4—thermocouple, 5—base; (**b**) bellow actuator where 1—bellow, 2—composite, 3—heater, 4—thermocouple, 5—mount of bellow, 6—base, 7—connecting wires; (**c**) heater.

Before casting, the composite components are first weighed in the correct amounts. In the first step, ethanol is added to compound A of silicon and mixed manually until a homogeneous mixture is obtained. Then compound B of silicon is added and mixed. In the case of bellow actuators, the heater is placed at the center of the bellow and then flooded with the liquid composite. While the composite is still liquid, a thermocouple is placed in a sheath inside it. The composite in the bellows was then left to solidify for 24 h at room temperature. After solidification of the composite, the bellow is placed in the circular recess of the base and then fixed with two plates, as is shown in Figure 2. For the tube actuator, the heater is placed into a mold in which it is poured. The mold has dimensions that correspond to the dimensions of the composite core. After 24 h, the composite core is pulled from the mold and placed in the tube. Then the composite core tube is fitted into the actuator base.

**Figure 3:**
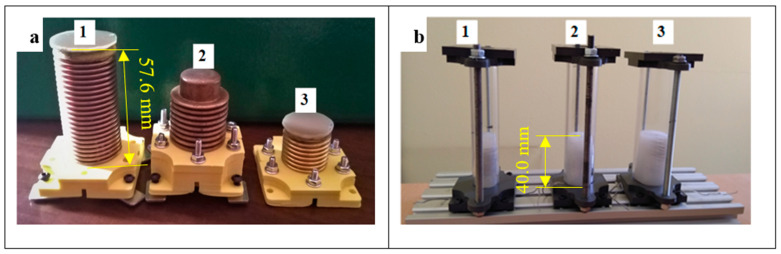
Photos of the bellow actuators investigated: (**a**) Bellow actuators: 1—big (B), 2—middle (M), 3—small (S); (**b**) tube actuators with equal initial length: 1—diameter of 15.5 mm, 2—diameter of 20.5 mm, 3—diameter of 25.0 mm.

The first investigations were carried out using three tube actuators shown in Figure 2a. They have a cylinder form with a composite inserted into polycarbonate tubes (Figure 3b). All actuators had double helical heaters embedded in the center (Figure 2c). The heaters were made of wire of resistive material FeCrAl 135 with a Teflon coating, which prevented accidental contact of the electrically powered wire with the bellows. The diameter of the wire was 0.4 mm.

Three tube actuators were prepared with different internal diameters, i.e., 15.5 mm, 20.5 mm, and 25.0 mm, with an identical initial composite length of 40 mm. Three more actuators with the same diameter but with equal composite volumes of 13.2 cm^3^ were also built. The dimensions and other parameters of the built actuators are given in Table 1.

The second set of designed and built actuators had the form of brass bellows filled with a composite in which the embedded helical heater was placed. Three bellows with different geometric parameters and different spring constants were used. The bellows and actuator parameters are given in Table 2. The thickness of the walls of all bellows was 0.2 mm.

In previous work on the functional properties of silicone/ethanol soft-actuator composites [11], the blocking force of transducers was examined in which the composite acted as a piston. However, the cylindrical composite samples tested had a constant diameter and different lengths. These were cylinders with a diameter of 20 mm and lengths of 25 mm, 50 mm, and 75 mm. Therefore, they also had a different volume, and this could have a major influence on the measured results. For this reason, in this research, we decided to investigate actuators with composite cores of equal volume. In addition, actuators with the same initial length but different volumes were also tested. The bellows for the actuators were selected from the ones available, so one had a volume comparable to the volume of composite cores in tube actuators of equal volume, one was smaller, and one was larger.

### 3.1. Test Stands

**Figure 4:**
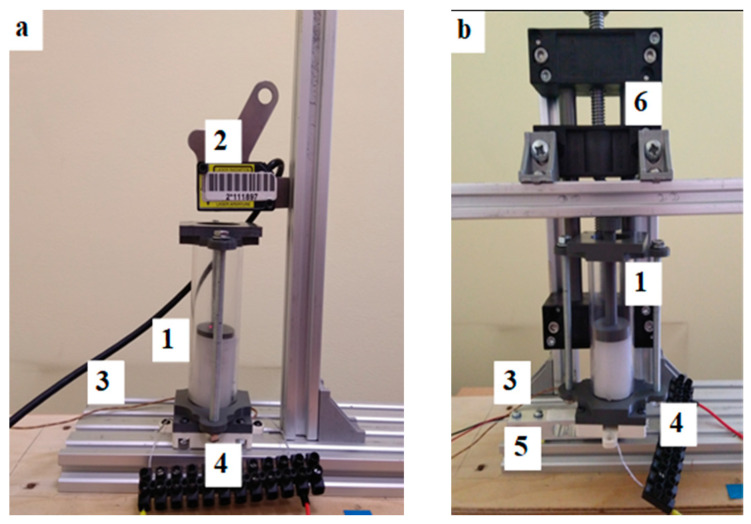
Test stand for measuring the displacement of the tube actuators (**a**) and the blocking force (**b**) for tube actuators: 1—actuator, 2—laser distance sensor, 3—thermocouple outputs, 4—connection of power supply, 5—tensometric beam, 6—blocking beam sliding mechanism.

**Figure 5:**
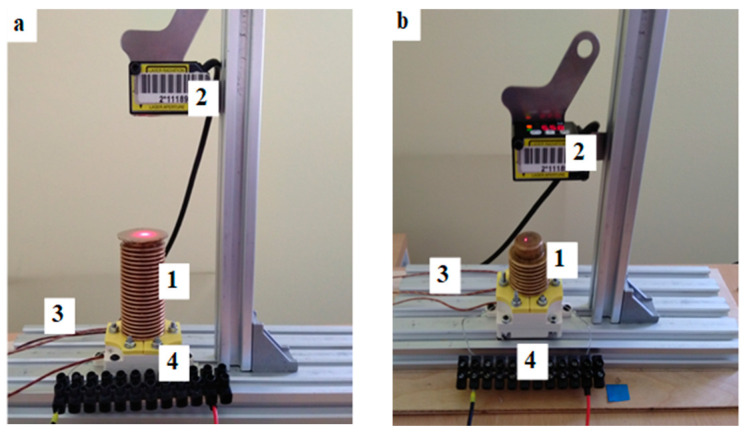
Stand for measuring elongation with bellow actuators: (**a**) bellow B, (**b**) bellow M, 1—actuator, 2—laser distance sensor, 3—outputs of thermocouples, 4—connection of power supply.

**Figure 6:**
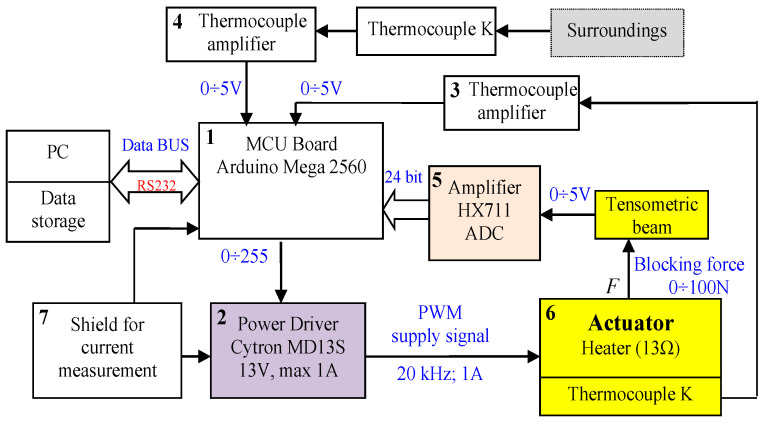
Block scheme of the test stand for measuring blocking force.

To perform investigations of all built actuators, two test stands were built. The first was used to measure the elongation during heating (Figure 4a) and the second was used to measure the blocking force (Figure 4b). In the stand measuring the elongation, a laser distance sensor was used. In the case of a unidirectional actuator on the face of the composite, a small plastic plate was placed to guarantee proper reflection of the laser beam. The analog signal from the sensor in the range of 0–5 V was transformed to digital form using a 15-bit AD converter and sent to the Arduino Mega 2560 microcontroller. The temperature of the composite was measured with K-type thermocouples. The signals from the thermocouples were amplified by dedicated amplifiers and sent to the microcontroller board. The amount of power that was used to heat the composite was regulated with the Cytron MD13S PWM power driver type. The current was measured using a digital shied with a sensor connected to the microcontroller Arduino board. This board was controlled by a PC, which received measured data (signals) for recording and visualization. The photo of the test stand for measuring elongation with bellow actuators is presented in Figure 5. For force measurement, the strain gauge (Figure 4b), mounted on a beam with a measuring range of 0–100 N, is used. It was connected to the dedicated signal amplifier type HX711. The actuator was mounted on the beam. The distance between the actuator front and the beam was regulated using the linear axis of the screw drive. In the case of the tube actuator, the force and movement were transmitted by the piston. The test stand for the force measurement and the scheme of its electronics is presented in Figure 4b and Figure 6 accordingly.

The control and measurement system for blocking force investigation presented in Figure 6 is built on the basis of an Arduino Mega 2560 microcontroller (1). The actuator heater is powered by the Cytron MD13S module (2), which generates the output signal to the heater using the PWM method. The input signal to the power controller is established by a control program implemented in the microcontroller. It sends data to the PC for storage and visualization. Two thermocouples are connected to amplifiers (3) and (4), which send the voltage signals of 0 V-5 V to the AD input of the Arduino module. To measure the actuator temperature, a type-K thermocouple was used. The second thermocouple with an amplifier (4) was used to measure the ambient temperature. For the measurement of actuator blocking force, the tensometric beam was used. Its analog output signal is given to the dedicated amplifier that also converts the signal from analog to digital (5). The tensometric beam has a measuring range of 0–100 N.

**Figure 7:**
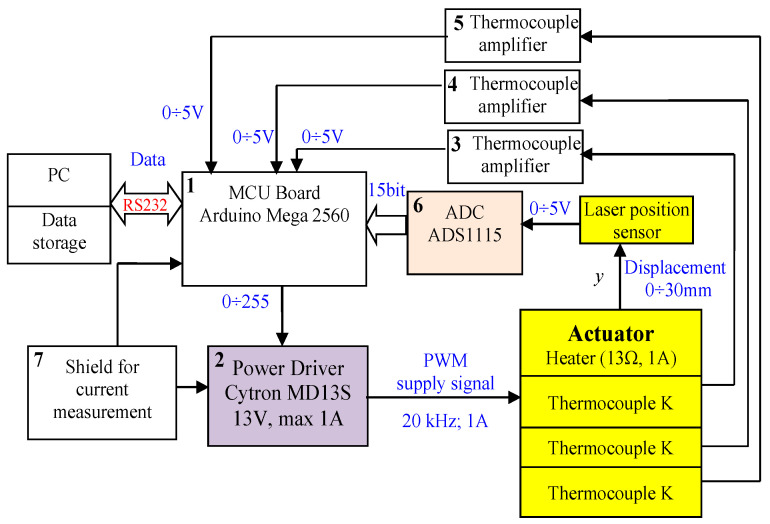
Block scheme of the test stand for distance and temperature measurement.

The control and measurement system for the investigation of elongation presented in Figure 7 is also built on the basis of an Arduino Mega 2560 microcontroller (1). In this case, three thermocouples are connected to amplifiers (3, 4, 5). For actuator displacement measurement, the Panasonic HG-C1050 laser distance sensor is used. The sensor has a measurement range equal to ±15 mm with a measurement center distance equal to 50 mm and repeatability of 30 µm. Its analog voltage output in the range of 0 V to 5 V is connected to a 15-bit analog-digital converter (6) with a reference voltage of 6.144 V. The resolution of the measurement is 1.13 µm. To measure the actuator temperature, the type-K thermocouple is used. The current is measured with the power shield (7). Information about its value is sent to the Arduino unit. The microcontroller also sends data to the computer for storage and visualization.

In the investigations, the responses of the actuators in the form of a generated displacement or force in time are measured. Additionally, an input signal, which is a current that supplies the heater, was measured. The current is switched on and had a constant value until the measurement inside the actuator temperature reached 75 degrees Celsius, and then the power is switched off. The feedback signal indicating the temperature inside the actuator came from a thermocouple located inside the actuator.

At the beginning of the investigations, only one thermocouple was used. However, it turned out to be insufficient for the recognition of the temperature in the whole composite. Therefore, to measure the temperature distribution in the composite, three thermocouples were applied. This enabled the measurement of how the heat flows from the heater to the composite space.

### 3.2. Preliminary Research

During preliminary investigations, the properties of composites with different amounts of ethanol were investigated. The purpose of these studies was to determine the content of ethanol in the composite, which will be used in future research. Three tube actuators with an internal diameter of 20.5 mm and an initial length of the composite equal to 40 mm were built. The ethanol contents in the prepared mixtures of silicon and ethanol were 5 vol.%, 10 vol.%, 20 vol.%, and 30 vol.%. It was observed that for 30 vol.%., there were problems with proper mixing. After the solidification of the composite, it was observed that some ethanol remains outside of the composite.

**Figure 8:**
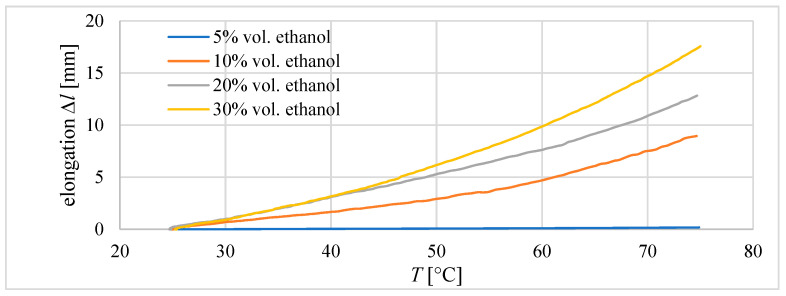
Elongation of the silicon–ethanol composite with different contents of ethanol during heating. The dimensions of the composite core were diameter *D* = 20 mm and initial length *l*_0_ = 40 mm.

In the first experiments, the elongation of the composite was measured during heating to 75 °C. The results are presented in Figure 8. The highest elongation value was achieved for the composite with 30% ethanol content. It was 17.5 mm and 43.75% of the initial length of the composite. For the compound with a content of 5% ethanol, only 0.2 mm of elongation was measured.

**Figure 9:**
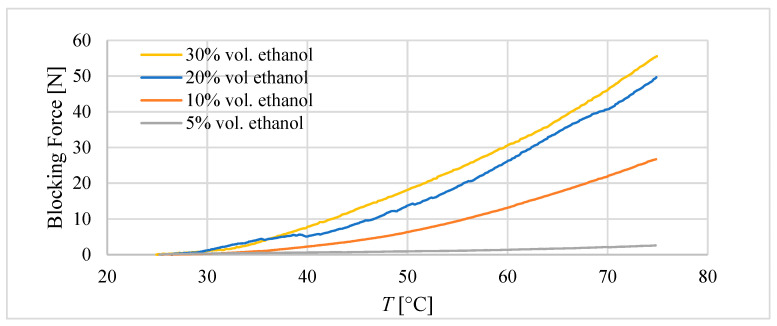
Blocking force of tube actuators based on a silicon-–ethanol composite with different content of ethanol. The dimensions of the composite core were diameter *D* = 20 mm and initial length *l_0_* = 40 mm.

In the second experiment, during heating to 75 °C, the movement of the composite was blocked and the blocking force was measured. The results are presented in Figure 9.

The highest value of blocking force was achieved for the composite with 30% ethanol at 55 N. However, it was only 5 N more than for the composite with the 20% ethanol content. Likely due to poor mixing, the real amount of ethanol that remained within the composite was lower than 30%.

Despite the better performance of the composite with 30 vol.% ethanol, because of difficulties in mixing it, for further investigation, the composite with 20 vol.% ethanol was chosen.

## 4. Results

A series of investigations were carried out on actuators to examine their elongation and generated blocking force. Before each test, the actuator temperature was approximately 24 °C. The heater was then turned on and powered until the composite temperature reached 75 °C. This prevented the composite from being damaged at higher temperatures because heating silicone-ethanol to a temperature above 85 °C leads to its rapid degeneration [11].

### Investigations of Tube Actuators

**Figure 10:**
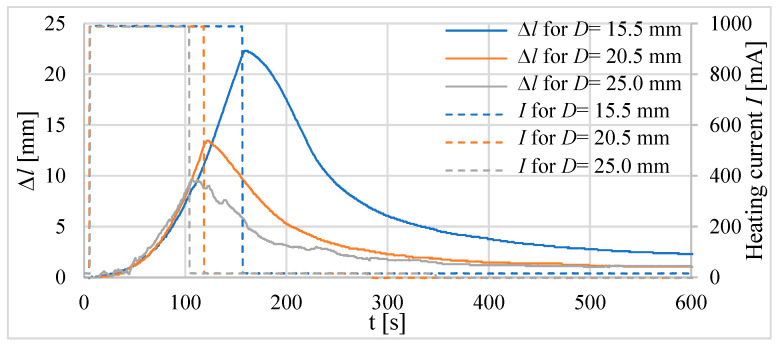
Elongation of tube actuators with equal volumes of composite and different diameters.

**Figure 11:**
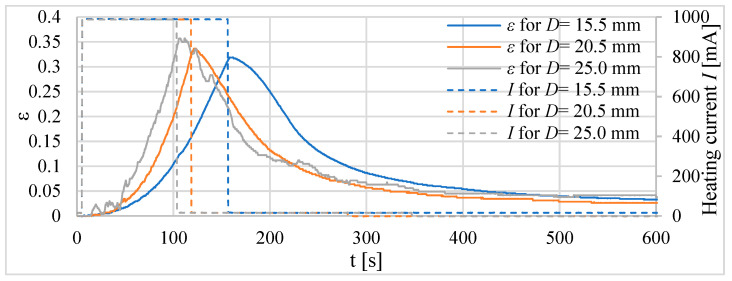
Relative elongation *ε* of tube actuators with equal volumes of composite and different diameters.

Firstly, three tube actuators with the same volume of the composite but different lengths were investigated. The signal waveforms of the absolute (Figure 10) and relative (Figure 11) elongations as a response to heating before reaching the temperature of 75 °C inside the actuator are recorded. The diameters of tube actuators are 15.5 mm, 20.5 mm, and 25.0 mm. The supply current equal to 1 A was switched on and off in steps. It was turned off when the temperature was 75 °C.

The longest heating time, that is, approximately 150 s, was needed for the actuator with the smallest diameter, that is, 15.5 mm of the composite core. It had a greater initial length, resulting in a greater tube area of heat exchange with the surrounding environment, resulting in the longest heating time. The actuator with a diameter of 20.5 mm needed 120 s to reach 75 °C. The heating time of the actuator with *D* = 25.0 mm was the shortest. The elongation, duration of heating to 75 °C, and areas of surface for the actuators are presented in Table 3. The largest elongation of 22.3 mm was obtained by a drive with a diameter of 15.5 mm.

**Figure 12:**
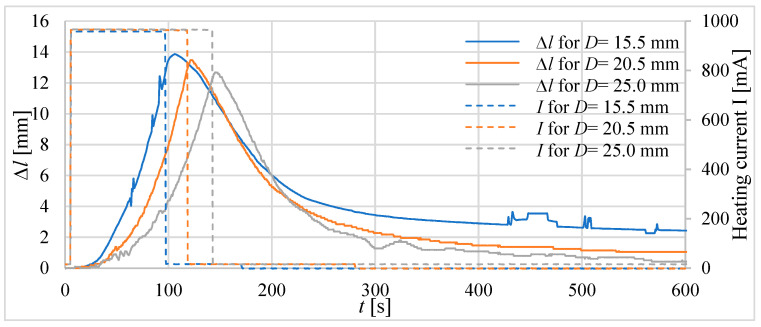
Absolute elongation for tube actuators of equal length and different diameters.

**Figure 13:**
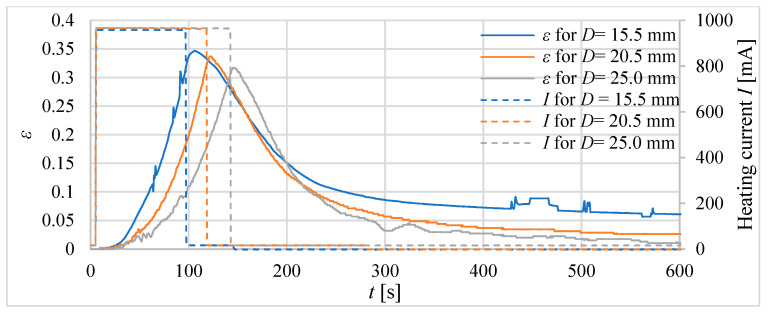
Relative elongation *ε* for actuators of equal length and different diameters.

In the second step of the investigations, tube actuators with an equal initial length of the composite, e.g., *l_0_* = 40 mm, were examined. The absolute and relative elongations (*Δl* and *ε*) of the actuators as a response to heating until the temperature reaches 75 °C inside the actuators are presented in Figure 12 and Figure 13.

For tube actuators, for most of the examined cases, after reaching the temperature of 75 °C, the relative elongation was between 0.26 and 0.36. The best results are obtained for the actuator with a diameter of 20.5 mm. The elongation for all tube actuators with equal length was almost the same, e.g., approximately 13 mm.

Equation (8) relates the elongation to the temperature rise. It was derived in [16] for an actuator without any housing. Therefore, the displacement of the composite was not blocked. This means that during heating, the actuator could expand in all six directions. In the case of tube actuators described here, the composite was placed in a tube closed on one side, thus the displacement was possible only in one direction. Moreover, the liquid composite was poured into the tube, in which it froze; therefore, it stuck firmly to the tube walls. Thus, the movement was limited by static friction between the composite and the inner wall of the tube. Therefore, Equation (8) must be corrected by adding the component describing the influence of friction on displacement. The increase in temperature results in an increase in the inner pressure of the composite, which increases the friction between the composite and the wall of a tube. This increase in inner pressure *Δp*(*t*) can be calculated with Formulas (1)–(3). Therefore, the equation below takes into account such a relationship

(11) $$\Delta l\left(t\right)=l_{0}\cdot \varepsilon_{zz}\left(t\right)-N_{F}\cdot \Delta p\left(t\right)$$

where *N_F_*—the coefficient [m/Pa] characterizing the influence of friction on elongation.

**Figure 14:**
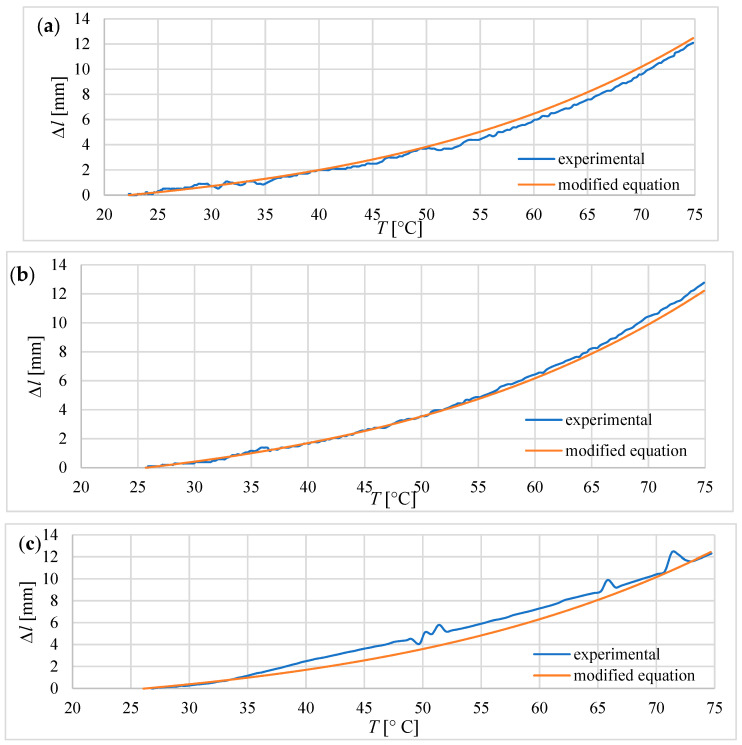
Experimental and calculated elongation during heating for tube actuators with different diameters and equal initial length *l_0_* = 40.0 mm. (**a**) elongation for tube actuator with *D* = 25.0 mm, (**b**) for *D* = 20.5 mm, (**c**) for *D* = 15.5 mm.

The experimental and calculated elongations with Equation (11) are presented in Figure 14. The coefficient *N_F_* was established as 0.165∙10^−6^ m/Pa.

In the blocking forces of the next step, the tube actuators were measured for actuators with equal volume and actuators with equal initial lengths. As in previous studies, during these tests, the composite was heated from 24 °C until it reached 75 °C in the center of the actuator. During each test, the elongation was blocked at a selected distance *Δl_b_*. The actuators were mounted on a tensometric beam that was used to measure the force.

**Figure 15:**
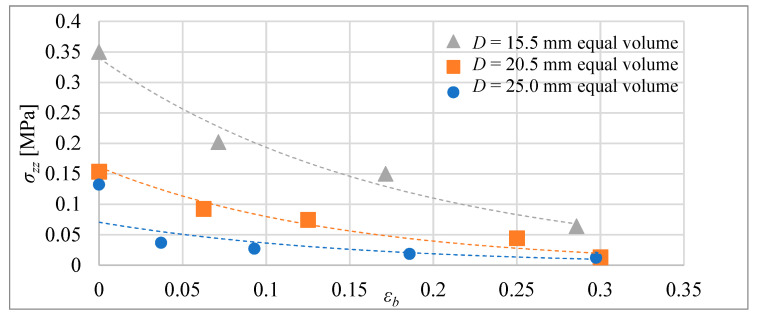
Blocking stress for tube actuators with equal volume for temperature of 75 °C in the center.

**Figure 16:**
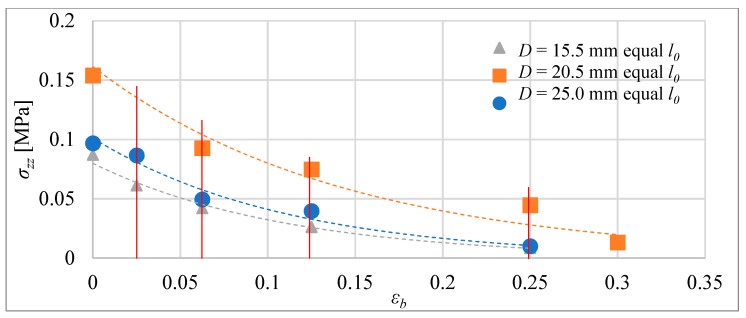
Blocking stress for tube actuators with equal initial length for 75 °C in the center.

In Figure 15, the absolute values of the blocking stress are shown as a function of the relative elongation of the actuators, with the same volume of the composite. The blocking stress for each relative elongation *ε_b_* = *Δl_b_*/*l*_0_ for the actuators was calculated by dividing the blocking force by the area of the cross-section of the composite core, i.e., *σ_zz_* = *F_b_*/*A*. In Figure 16, similar results are presented, but for actuators with equal initial lengths. The geometrical figures, i.e., triangle, square, and circle, represent force-measuring points, i.e., elongation, which are established before heating by setting the blocking element. This element made it impossible to extend the composite beyond the set distance.

In the following investigations, bellow actuators were subjected to tests similar to those described above for tube actuators.

**Figure 17:**
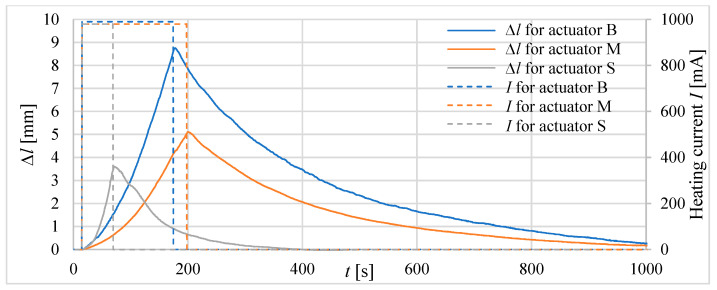
Absolute elongation of the bellow actuators heated to 75 °C.

**Figure 18:**
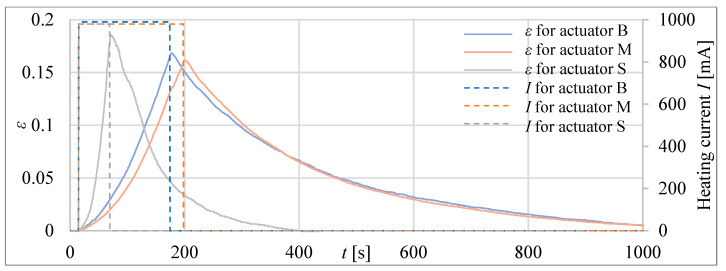
Relative elongation for bellow actuators.

At first, the actuator elongations were tested by heating to 75 °C and then cooled in steady ambient air. The results are presented in Figure 17 and Figure 18.

For the bellow actuators investigated, the relative elongation at a temperature of 75 °C ranged between 15% and 20%. This is less than for tube actuators, which can elongate to 36%. This is the consequence of the influence of the spring forces of the bellows. The bellow actuators are also slower than the tube actuators when they are heated under similar conditions (compare curves in Figure 11 and Figure 18). The heating times are presented for comparison in Table 4, where it is visible that the bellow actuator with a similar volume to the tube actuator needed more time during heating to reach 75 °C. The bellow actuators act slower, not only because of the influence of the spring force of the metal bellow but also because their shapes are bigger, which resulted in greater dissipation of heat to the atmosphere during heating.

**Figure 19:**
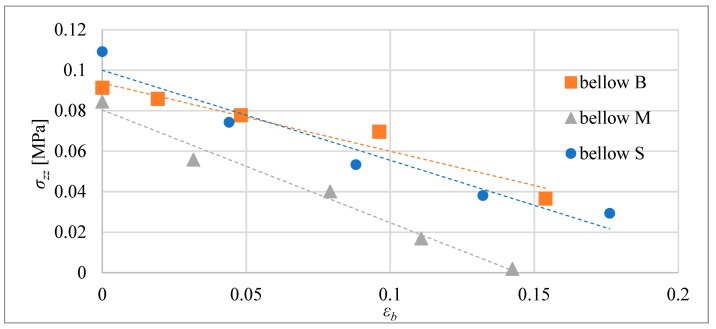
Blocking stress of actuators as a function of relative elongation for a temperature of 75 °C.

In the next part of the research presented here, the blocking force of the bellow actuators was investigated. The dependence of blocking stress as a function of relative elongation is presented in Figure 19 Similar to tube actuator tests, the composite was heated until it reached 75 °C in the center of the actuator. During each test, elongation was blocked at a selected distance *Δl_b_*. The actuators were mounted on a tensometric beam that was used to measure the force. The blocking stress *σ_zz_* for each relative elongation of the actuators was calculated by dividing the blocking force by the effective area of the cross-section of the bellow actuator.

**Figure 20:**
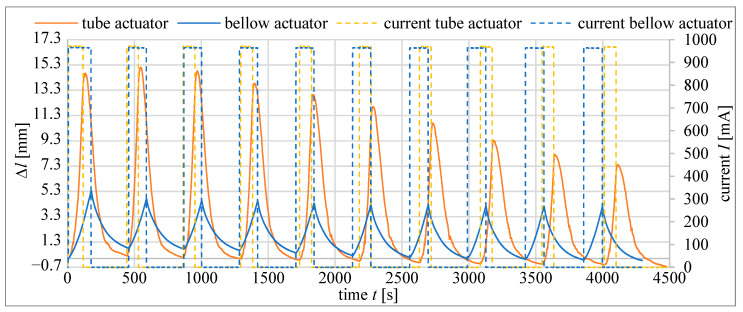
Cyclic work of the tube actuator (*D* = 20.5 mm, *l*_0_ = 40.0 mm) and the bellow actuator S.

**Figure 21:**
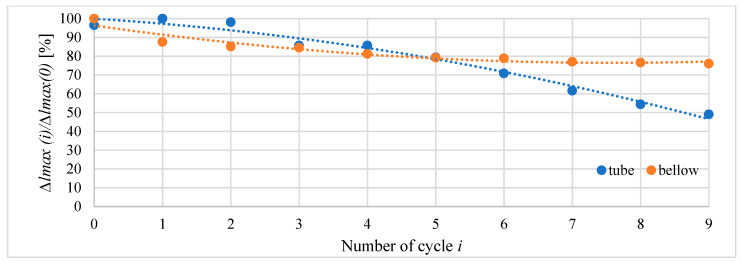
Composite wear in tube and bellow actuator.

For better recognition of actuators’ behavior in cyclic work, further tests were performed. The actuators were heated until they reached 75 °C, then cooled to 50 °C and then again heated to 75 °C, etc. This was repeated 10 times. To speed up the tests, the stand was equipped with a fan to accelerate cooling. Each time the fan was turned on, it ran at the same speed when cooling the actuator. Figure 20 presents the curves recorded during these cycles for the bellow actuator type M and the tube actuator with an inner diameter of 20.5 mm and an initial length of 40 mm. Furthermore, the maximal actuator elongation changes with the number of cycles are presented. The results show the main advantage of the bellow actuator, which is its better durability compared to tube actuators. For tube actuators, their performance becomes worse with every cycle. In Figure 21, it can be seen that after the first few cycles, the maximal elongation of the bellow actuator stabilizes, while for the tube actuator it consistently decreases. As can be seen, the bellow actuator completed 10 cycles in approximately 180 s faster than the tube actuator. This is mainly due to faster cooling.

The reduction of maximal elongation during cyclic heating to 75 °C and cooling for the tube actuator (*D* = 20.0 mm, *l*_0_ = 40.0 mm) and the bellow actuator type S is shown in Figure 18. The first cycle number 0 started at room temperature. The next started at 50 °C. The wear of the bellow actuator is much less than the wear of the tube actuator, of which elongation is reduced by 50% after only 10 cycles. For the same number of cycles, the elongation of the bellow actuator was reduced by 25%. However, after the seventh cycle, the value of maximal elongation stabilizes and stays at the same level even for several hundred cycles.

## 5. Conclusions

In this paper, two types of actuators with a silicon–ethanol composite are built and investigated. In the first one, a plastic tube was used as housing, and in the second one, the metallic bellow is applied. Finally, six tube actuators and three bellow actuators were designed and built. Moreover, during the preliminary tests, it was found that the generated forces and elongations increase with increasing ethanol content. However, with a 30 vol.% content of ethanol, mixing becomes difficult.

The test conditions for the actuators included heating the composite until it reached 75 degrees Celsius. In that condition, tube actuators were able to achieve maximal relative elongation between 35% and 32%. That is less than was predicted according to Equation (8) for the elongation of the non-blocking composite, proposed in a previously published paper [16]. Therefore, the corrections were introduced into these equations, taking into account the presence of frictional forces between the composite and the tube, which reduces free elongation.

For the bellow actuators tested, maximal relative elongation for conditions of the investigation were 19%, 16%, and 17% according to the actuator types marked S, M, and B. The reduction of maximal elongation in comparison with tube actuators results from the influence of the spring force of the bellow that counteracts elongation. Moreover, the maximal blocking stress obtained for bellow actuators that ranged between 0.08 and 0.11 MPa was less than for tube actuators, where it was between 0.1 and 0.35 MPa. However, the main advantage of bellow actuators in comparison to tube actuators is less susceptibility to rapid degradation. Their properties stabilize after a few cycles; therefore, they can be used many times without wear.

The fast degradation of composites in tube actuators during cyclic work suggests that this material should be improved with an additional outer layer with a lower permeability to ethanol vapors.

Equations (4), (9), and (10) given in a previous paper on Soft actuators based on liquid–vapor phase change composites [4], which describe the elongation and blocking stress of the composite, can be a good base for the formulation of equations predicting the behavior of actuators during heating in the range of 24 °C–75 °C. However, they need modifications specific to the particular type of actuators. In the case of tube actuators, it is necessary to take into account the influence of friction between the composite core and the tube wall. For equitation 10, in which the modification was implemented, the results of calculated elongations during heating and the results measured during the experiments were consistent. In the case of bellow actuators, the significant influence on the behavior of the actuator concerns the properties of the bellow.

Further research will investigate the influence of the bellow parameters on the performance of the actuator based on the silicone–ethanol composite.

There is a possibility of using the composite in self-decomposing structures, infusion pumps, or over-temperature sensors. In the case of infusion pumps, where slow drive operation is required, a low speed of actuation of the silicon–ethanol composite could be its advantage. However, for this application, further testing of the durability and repeatability of the composite is required. In the case of over-temperature sensors, the ability to generate forces and displacements depending on the increase in temperature could be used.

## Figures and Tables

**Table 1:** Dimensions of Composite Cores used in Tube Actuators.

	Diameter *D* [mm]	Initial Length *l*_0_ [mm]	Volume *V* [cm^3^]
Equal initial length	15.5	40.0	7.6
	20.5	40.0	13.2
	25.0	40.0	19.6
Equal volume	15.5	70.0	13.2
	20.5	40.0	13.2
	25.0	26.9	13.2

**Table 2:** Parameters of bellows actuators.

Bellow	Volume *V*	Spring Constant *k*	Initial Length *l*_0_	Minimal Diameter *D_min_*	Maximum Diameter *D_max_*	Effective Cross Section *A*	Number of Segments *n*
	cm^3^	N/mm	mm	mm	mm	mm^2^	—
B	16.95	1.30	57.6	17	26.4	369.8	22
M	13.54	5.53	48.5	19.2	27	419.1	9
S	4.15	0.41	19.4	12	22.7	237.8	7

**Table 3:** The parameters and results of tube actuators with equal volume.

Diameter [mm]	Heating Time to 75 °C [s]	Total Surface Area [cm^2^]	Lateral Surface Area [cm^2^]	Maximal Elongation *Δl_max_* [mm]	Maximal Relative Elongation *ε*
15.5	151	37.8	34.1	22.3	0.32
20.5	113	32.4	25.8	13.5	0.34
25.0	102	30.9	21.1	9.6	0.36

**Table 4:** Comparison of selected actuators. All actuators were heated with a current of approximately 1 A with heaters with this same resistance.

Actuator	Volume of the Composite [cm^3^]	*t* to Reach 75 °C [s]	*t* to Achieve 5.0 mm [s]
Bellow M *l*_0_ = 48.5 mm	13.54	198	198
Tube diameter 20.5 mm, *l*_0_ = 40 mm	13.2	113	86

## Data Availability

Not applicable.
